# Comparison of McMaster and FECPAK^G2^ methods for counting nematode eggs in the faeces of alpacas

**DOI:** 10.1186/s13071-018-2861-1

**Published:** 2018-05-02

**Authors:** Mohammed H. Rashid, Mark A. Stevenson, Shea Waenga, Greg Mirams, Angus J. D. Campbell, Jane L. Vaughan, Abdul Jabbar

**Affiliations:** 1 Department of Veterinary Biosciences, Melbourne Veterinary School, The University of Melbourne, Werribee, Victoria 3030 Australia; 2 Techion Group Limited, PO Box 5057, Moray Place, Dunedin, 9058 New Zealand; 3 Cria Genesis, PO Box 406, Ocean Grove, Victoria 3226 Australia

**Keywords:** McMaster technique, FECPAK^G2^, Nematodes, Faecal egg count, Alpaca

## Abstract

This study aimed to compare the FECPAK^G2^ and the McMaster techniques for counting of gastrointestinal nematode eggs in the faeces of alpacas using two floatation solutions (saturated sodium chloride and sucrose solutions). Faecal eggs counts from both techniques were compared using the Lin’s concordance correlation coefficient and Bland and Altman statistics. Results showed moderate to good agreement between the two methods, with better agreement achieved when saturated sugar is used as a floatation fluid, particularly when faecal egg counts are less than 1000 eggs per gram of faeces. To the best of our knowledge this is the first study to assess agreement of measurements between McMaster and FECPAK^G2^ methods for estimating faecal eggs in South American camelids.

**Electronic supplementary material:**

The online version of this article (10.1186/s13071-018-2861-1) contains supplementary material, which is available to authorized users.

## Letter to the Editor

Parasitic gastroenteritis caused by gastrointestinal nematodes (GINs) is responsible for significant clinical and subclinical problems in domesticated South American camelids (SACs), alpacas and llamas, resulting in economic losses arising from lowered production of fibre, meat and/or leather [1, 2]. Although no drugs are registered for use against GINs of SACs in Australia, anthelmintics are routinely used to deworm alpacas and llamas, mostly without assessment of worm burdens prior to treatment [3].

In SACs, the burden of GINs can be assessed using various coprological methods originally developed for domestic ruminants [4], with estimation of the number of GIN eggs per gram of faeces (faecal egg count, FEC). The most commonly used coprological method is the McMaster technique [5, 6]. The choice of floatation solution (saturated sugar or salt) is important for this technique because the type and specific gravity of floatation solutions can affect FEC results [5]. Previously, saturated sugar was found to be superior to salt as a floatation solution for the detection of some GINs in SACs as the rate of water loss and distortion of GIN eggs was slower using sugar [6, 7]. Although the McMaster technique has stood the test of time because it is a relatively simple and cheap procedure to carry out, it has a number of disadvantages, including difficulties identifying eggs when faeces are thick and dark, and high levels of technical skill and experience are required to identify and count nematode eggs of different species [5]. To overcome these limitations, other FEC estimation methods such as Kato-Katz®, FLOTAC® and FECPAK have been developed and validated in both humans and animals [5, 8, 9]. Recently, smartphone applications have been found to provide a more efficient approach for counting parasite eggs in the faeces of animals compared with the McMaster technique [10].

The FECPAK method is based on a modified McMaster technique with a minimum detection limit of 30–35 eggs per gram (EPG) of faeces [11]. The original FECPAK method was developed in New Zealand to provide a simple on-farm method for FEC estimation. The updated FECPAK^G2^ method uses a floatation-dilution approach similar to the McMaster technique, but involves capturing digital images of samples without the use of a microscope. Digital images of samples are stored, ready for assessment by trained technicians for identification and counting nematode eggs [12]. Each digital image remains available for reference and auditing purposes. Setting up the FECPAK^G2^ test does not require specialised laboratory equipment or technical skills, and preparation can be done easily in the field by a lay operator. As a result, large numbers of samples can be processed at one time and images analysed later.

The aim of this study was to compare FEC estimates of GINs in alpacas using FECPAK^G2^ and the McMaster technique using two floatation solutions (saturated sodium chloride and sucrose solutions).

Defining *α* = 0.05, a study power of 0.80 and agreement limits of ± 2100 EPG, we estimated that a sample size of 94 faecal samples was required to assess agreement between the two methods of measurement [13]. Briefly, fresh faecal samples (*n* = 94) were collected directly from the rectum of 3-month to 16-year-old Huacaya alpacas, from commercial farms (*n* = 10) in New South Wales, Queensland and Victoria, Australia. Samples were stored at 4 °C for up to seven days until the time of testing.

Each faecal sample was tested using modified McMaster technique [4, 6]. Briefly, four grams of faeces were soaked for 5–30 minutes in 11 ml of water in 60 ml plastic containers. The faecal slurry was then mixed with either 45 ml of saturated sodium chloride [specific gravity (SG) 1.20, Merck, Germany] or white sugar (SG 1.27, www.csrsugar.com.au) solution and homogenised using a metal spatula. After 30–45 minutes, a sample was drawn from the suspension using a sieve-top pipette (sieve aperture size 12 meshes per cm). Following agitation, the sample was introduced into two chambers of a Whitlock egg counting slide (www.whitlock.com.au) which was then placed on the stage of a compound light microscope. After five minutes, eggs were counted. The minimum detection limit using this method was 15 EPG.

All of the faecal samples that were processed using the McMaster technique were then processed using the FECPAK^G2^ (Techion Group Ltd., New Zealand; www.techiongroup.com) method using salt and sugar solution as per the manufacturer’s protocol. Four grams of faeces were selected for each sample.

The arithmetic means of EPG were calculated for faecal egg counts obtained using the two methods and differences in arithmetic means were tested using the Wilcoxon signed rank test. A *P*-value < 0.05 was considered as statistically significant. Differences in EPG estimates assessed using the McMaster and FECPAK^G2^ methods using sugar and salt solutions were statistically significant (*P*-values 0.003 and 0.001 for salt and sugar solutions, respectively). Due to the highly skewed distribution of the FEC data, individual FECs were log transformed [log_10_(EPG + 15)] [14], and agreement of the FEC estimates using the McMaster and FECPAK^G2^ methods assessed using the Lin’s concordance correlation coefficient [15] and Bland-Altman plot [16]. To provide interpretable Bland and Altman plots, limits of agreement for the log-transformed data were calculated and transformed back to the original scale using the approach described by Euser et al. [17]. These limits of agreement were plotted on the original scale using conventional Bland and Altman plots. Statistical analyses were carried out using the *epiR* package [18] implemented in R [19].

A total of 64–89% faecal samples was test-positive using the McMaster and FECPAK^G2^ methods (Table 1). However, when saturated salt solution was used, more samples had at least more than one EPG using the McMaster technique (89%; 81/91) compared with FECPAK^G2^ (77%; 70/91). Using sugar solution, more samples had at least more than one EPG using the McMaster technique (73%; 69/94) compared with FECPAK^G2^ (64%; 60/94; Table 1). There was a significant difference (*P* = 0.03) in the proportions of positive samples tested by both methods when salt solution was used (Table 1).

**Table 1:** Descriptive statistics of the untransformed faecal egg counts of alpaca gastrointestinal nematodes using McMaster and FECPAK^G2^ methods

Floatation solution	Method	% Test-positive samples (proportion)	*P*-value	Arithmetic mean EPG ± SE	95% CI	Range of EPG
Salt	McMaster	89 (81/91)	0.03	335 ± 62	211–458	0–3435
	FECPAK^G2^	77 (70/91)		438 ± 83	273–603	0–5180
Sugar	McMaster	73 (69/94)	0.16	448 ± 138	174–723	0–10,515
	FECPAK^G2^	64 (60/94)		280 ± 86	109–450	0–6930

*Abbreviations*: *EPG* eggs per gram of faeces, *SE* standard error of the mean, *CI* confidence interval

**Fig. 1:**
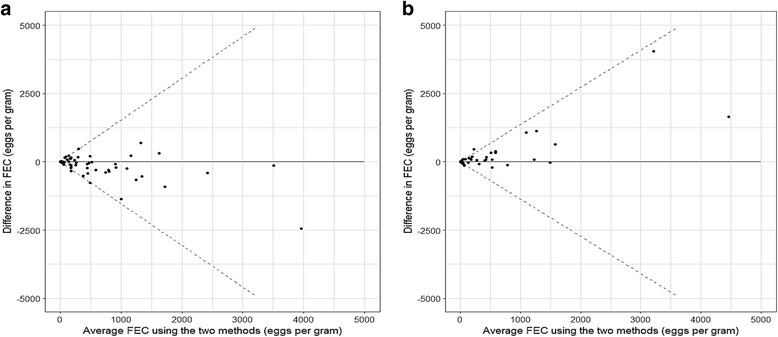
Bland-Altman agreement plots for McMaster and FECPAK^G2^ methods. The dashed lines on each plot show the back-transformed limits of agreement using salt solution (**a**) and sugar solution (**b**)

Lin’s concordance correlation coefficient was greater when sugar solution was used as a floatation fluid compared with salt (0.84, 95% CI: 0.77–0.89 and 0.78, 95% CI: 0.68–0.85, respectively) (see Additional file 1). These metrics are supported by the Bland-Altman plots shown in Fig. 1 where FEC differences for sugar are more tightly clustered around zero (Fig. 1b) compared with salt (Fig. 1a), particularly when mean EPGs were less than 1000. For the relatively small numbers of samples where mean EPGs were greater than 2500, differences in the two methods were greater for sugar (Fig. 1b).

To the best of our knowledge this is the first study to assess agreement between SAC FECs estimated using the McMaster and FECPAK^G2^ methods. Our results show moderate to good agreement between the two methods, with better agreement achieved when saturated sugar is used as a floatation fluid, particularly when FECs are less than 1000 EPG. The advantages of the FECPAK^G2^ method are that it does not require specialised laboratory equipment or highly trained staff on farm, and images are stored online for perpetuity.

## Additional file

Additional file 1: **Figure S1.** Concordance correlation coefficient (CCC) plots showing line of perfect concordance (dotted line) and estimated concordance (solid line) between McMaster and FECPAK^G2^ methods using salt solution (**a**) and sugar solution (**b**). (TIF 520 kb)

## Abbreviations

CCC: Concordance correlation coefficient
EPG: Eggs per gram of faeces
FEC: Faecal egg count
GINs: Gastrointestinal nematodes
SACs: South American camelids
