# Supplementary figures and images for: Comparison of McMaster and FECPAKG2 methods for counting nematode eggs in the faeces of alpacas
Source: Parasit Vectors. 2018 May 2;11:278. doi: 10.1186/s13071-018-2861-1 (PMC5930814; doi:10.1186/s13071-018-2861-1)

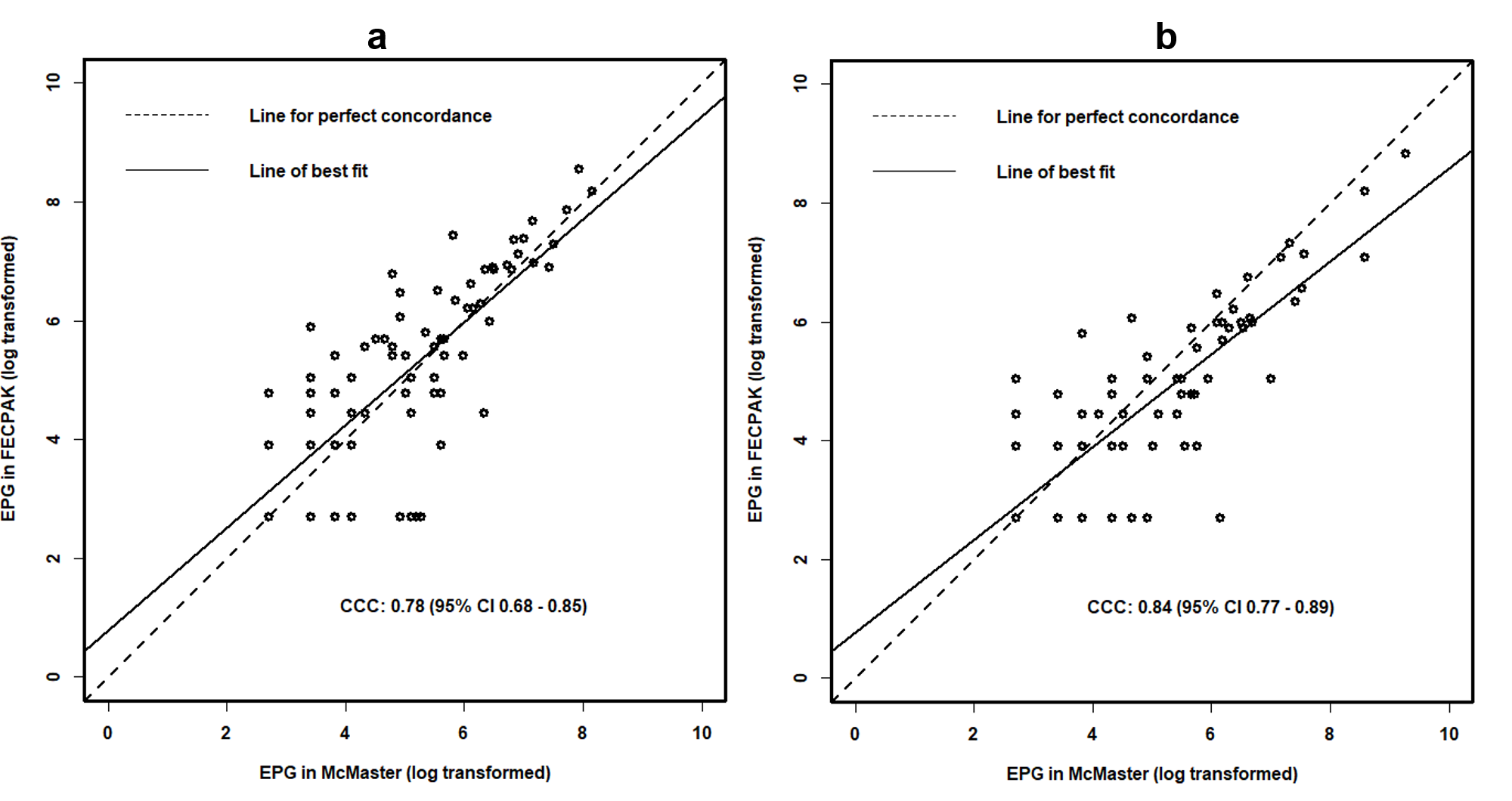

Supplement: Supplementary file 1 — Figure S1. Concordance correlation coefficient (CCC) plots showing line of perfect concordance (dotted line) and estimated concordance (solid line) between McMaster and FECPAKG2 methods using salt solution (a) and sugar solution (b). (TIF 520 kb) [file 13071_2018_2861_MOESM1_ESM.tif]
